# Verbal communication in tracheostomised mechanically ventilated patients leads to improved respiratory mechanics

**DOI:** 10.1186/2197-425X-3-S1-A314

**Published:** 2015-10-01

**Authors:** A-L Sutt, L Caruana, P Cornwell, K Dunster, C Anstey, J Fraser

**Affiliations:** The Prince Charles Hospital, Critical Care Research Group, Brisbane, Australia; University of Queensland, School of Medicine, Brisbane, Australia; The Prince Charles Hospital, Chermside, Australia; Griffith University, Brisbane, Australia; Sunshine Coast Hospital and Health Service, Intensive Care Unit, Nambour, Australia

## Introduction

Due to voicelessness, communication is often a source of extreme frustration for tracheostomised mechanically ventilated patients. Cuff deflation is required to enable speaking valve (SV) use and has been thought to potentially cause derecruitment of the patients' lungs. Diaphragm weakness is also known to develop rapidly whilst patients are mechanically ventilated. Recently, data was reported that with SV in-situ end-expiratory lung volumes (EELV) increased. However, there are no published data on ventilation distribution or the potential effect of SV on the diaphragm.

## Objectives

To assess EELV distribution and abdominal to chest ratio when using a SV in-line with mechanical ventilation circuit of tracheostomised ICU patients.

## Methods

Twenty consecutive tracheostomised cardio-thoracic ICU patients weaning off mechanical ventilation and using an in-line SV were recruited. Ten patients were receiving Pressure Support Ventilation and 10 were on High Flow Tracheostomy piece. All patients were monitored using Electrical Impedance Tomography and Respiratory Inductance Plethysmography pre, during and post 30min of SV use. Outcome measures included EELV distribution and abdominal:chest ratio.

## Results

The patients showed increased EELV with a significant increase (p < 0.001) in all regions (522 in anterior, 299 posterior, 180 in left and 641 right) of the lungs during 30min with SV in ventilation circuit. EELV continued to increase in all regions once SV was removed (1505 globally, p < 0.001). This increase in EELV happened irrespective of the patients'ventilation status. There was a significant increase in abdo:chest ratio (p = 0.03) indicating increased abdominal mobility suggestive of improved diaphragm activity with SV.

## Conclusions

SV use in this cohort of tracheostomised mechanically ventilated cardio-thoracic ICU patients resulted in likely improved recruitment of the patients' lungs with increased diaphragm activity. More research is needed to determine whether this improvement in patient communication could also lead to shorter ventilator weaning times, and improved ICU outcomes.

## Grant Acknowledgment

The Prince Charles Hospital Foundation

